# Carbonized Polymer Dots: Influence of the Carbon Nanoparticle Structure on Cell Biocompatibility

**DOI:** 10.1021/acsomega.4c05011

**Published:** 2024-09-05

**Authors:** Mayara Martins Caetano, Amanda Blanque Becceneri, Marcos Vinícius Ferreira, Rosana Maria Nascimento Assunção, Roberto Santana da Silva, Renata Galvão de Lima

**Affiliations:** † Instituto de Química, Universidade Federal de Uberlândia, Avenida João Naves de Ávila, 2121-Bairro Santa Mônica, Uberlândia, Minas Gerais 38304-402, Brazil; ‡ Instituto de Ciências Exatas e Naturais Do Pontal, ICENP, Universidade Federal de Uberlândia, Rua Vinte, 1600, Tupã, Ituiutaba, Minas Gerais 38304-402, Brazil; § Faculdade de Ciências Farmacêuticas de Ribeirão Preto, USP, Avenida Do Café S/n, Vila Monte Alegre, Ribeirão Preto, São Paulo 14040-903, Brazil

## Abstract

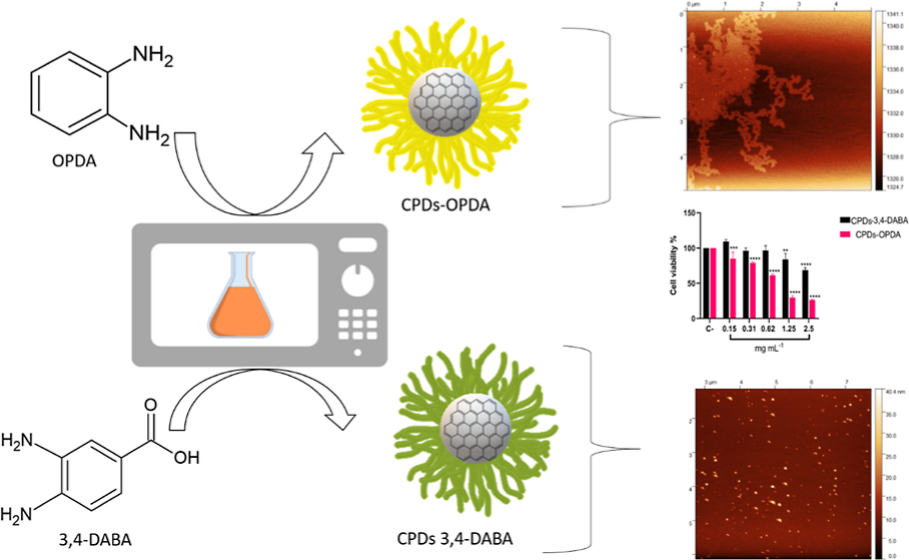

Carbonized polymer dots (CPDs) were obtained by using microwave irradiation under the same conditions. However, different carbogenic precursors were used, such as aromatic diamine molecules, *ortho*-phenylenediamine (*o*-OPDA), and 3,4-diaminobenzoic acid (3,4-DABA). Both carbon nanoparticles showed different structural results based on Fourier transform infrared spectroscopy, Raman spectroscopy, X-ray diffraction, and atomic force microscopy analyses. However, there are similar spectroscopic (UV–visible and fluorescence emission) profiles. The photophysical results, like quantum yield (QY) and fluorescence lifetime, were not identical; CPDs-OPDA has a higher QY and fluorescence lifetime than CPDs-3,4-DABA. CPDs-3,4-DABA presents a more hydrophobic character than CPDs-OPDA and has a more negative superficial charge. Cell viability studies in both standard and tumor lines demonstrated higher cytotoxicity from CPDs-OPDA than that from CPDs-3,4-DABA. The oxidative stress identified in cells treated with CPDs-OPDA was based on reactive oxygen species and associated with nitric oxide production. CPDs-3,4-DABA showed more DPHH inhibition than CPDs-OPDA, indicating the antioxidant activity of CPDs.

## 1 Introduction

Significant advances have recently been achieved in the research field of carbon-based nanoparticles, which are named carbon dots (CDs) based on their molecular structure.^1−7^

Generally, CDs are synthesized based on low-cost natural sources^8,9^ and are classified according to the synthesis mechanisms, micro-/nanostructures, and spectroscopic properties.^10^ Maybe the high-fluorescence quantum yield (QY) presented by CDs is the most commonly explored property, which can be attributed to the formation of different structures during polycondensation and carbonization processes of precursors, including different conjugation domains,^11^ graphitization in the carbon core,^12^ functionalization degree on the surface,^13^ specific molecular structures from polymerization of small molecules,^14^ and degree of cross-linking.^12^

CDs are zero-dimensional nanoparticles with a size of less than 10 nm. Since their discovery in 2004,^15^ varieties of fluorescent carbon nanoparticles have been described in the literature, including graphene quantum dots (GQDs), carbon quantum dots (CQDs), carbon nanodots (CNDs), polymer CDs (PCDs), and carbonized polymer dots (CPDs), which are classified according to the specific carbon core structure and surface groups (functional groups or polymer molecular state).^16^

The structure of CDs consists of a sp^2^/sp^3^ carbon skeleton and oxygen-/nitrogen-based groups.^17^ Top-down synthesis typically produces CDs with a graphite-type carbon skeleton, identified as GQDs or CQDs.^18^ GQDs are small graphene fragments consisting of a single or few graphene sheets with obvious graphene lattices and chemical groups on the edge or within the interlayer defect. These contribute to unique properties, such as the quantum confinement and edge effects.^16^ CQDs have a core with different graphite structures connected to surface functional groups.^19^ Most of the classification of CQDs arises from powder X-ray diffraction (XRD) analysis. CQDs are characterized by a broad peak close to θ = 25° in XRD^2^. The typical CQD Raman spectral sp^2^ and sp^3^ carbon signals can be ascribed to D and G bands.^20^ The photoluminescence mainly originates from the defect/surface state and subdomain state within the graphitic carbon core without the quantum confinement effect of the particle size and crystal lattice.^17^ CNDs are always spherical. They are obtained from a top-down process and usually have the inner hybridization of sp^2^ and the outer hybridization of sp^3^. These hybridized carbon structures tend to have functional groups containing oxygen atoms.^21^

Yang et al. reported in 2017^22^ that some starting materials cannot undergo total carbonization during the bottom-up synthetic process. In this case, the carbon nanoparticles obtained were classified as PCDs. PCDs differ from traditional carbonized CDs regarding their chemical structure, properties, and fluorescence origin. PCDs may not inherit all properties of CDs, such as the crystallized graphite structure, quantum size effect, and stability against photobleaching. The formation of PCDs depends on the degree of carbonization, which is low, and, in some instances, carbonization does not even happen. However, the nanosized dots are still formed during the synthesis.

Researchers have recently attempted the chemical synthesis of CDs starting from molecules or polymer precursors using the bottom-up procedure.^23−25^ In 2019, Yang et al.^16^ described the evolution of CD synthesis. It defined the term CPDs as nanoparticles characterized by a polymer/carbon hybrid structure rather than the structure of the carbon main body, which differs from traditional carbonized CDs or PCDs. The CPDs possess prominent optical properties that originate from the properties of the polymer.

Nagao et al.^26^ and Yang et al.^24^ propose that CPDs possess the characteristics of both carbon materials and organic luminescent materials with carbonized and cross-linked polymer hybrid nanostructures.

Most CDs prepared through the bottom-up synthesis method from asymmetrical precursors belong to the CPDs category.^27^ The CPDs present a special “core shell”. The core has a highly cross-linked rigid polymer network with slight carbonization, while the outer shell contains many small polymer chains with large functional groups.^23^

Among the carbon-based nanosystems, CDs have attracted attention mainly due to the possibility of application in biological areas.^28−30^ According to the literature, the toxicity of CDs is not related to surface modification but rather to the surface charge and the role of chemical groups on the surface limited by this charge.^31^ In addition, recent studies have shown that the culture medium’s size, charge, and aggregation can influence the toxicity of CDs.^32^

Zboril et al.^32^ prepared three CDs with differing surface functionalization: pristine CDs (CDs-Pri) with a negative charge, −28 mV, due to carboxylic groups, polyethyleneglycol-modified dots with a neutral charge, −6 mV, (CDs-PEG), and polyethyleneimine-coated dots with a positive charge, +53 mV, (CDs-PEI). Based on measurements of the viability of the cell and flow cytometry, it was demonstrated that positive charge CDs-PEI entered the cell nucleus. Negative charge CDs-Pri stimulated cell proliferation and led to oxidative stress, and neutral CDs-PEG showed lower cytotoxicity (IC_50_ = 300 μg mL^–1^)

In addition, recent studies have shown that the size and charge of the CDs cannot alter the cell viability under higher concentrations.^33,34^ However, the aggregation in the culture medium and cell phase can influence the toxicity of the CQDs. Wang et al. proposed a purpose for two kinds of CDs with surface passivation by 3-ethoxypropylamine (EPA-CDs) and oligomeric polyethylenimine (PEI-CDs). In media containing serum, the cellular uptake of PEI-CDs was overall lower, probably due to the formation of a protein corona on the dot surface, which would impede the entry of PEI-CDs into cells. EPA-CDs cultured in media with serum exhibited a higher cellular uptake than that in serum-free media.^35^

**Figure 1:**
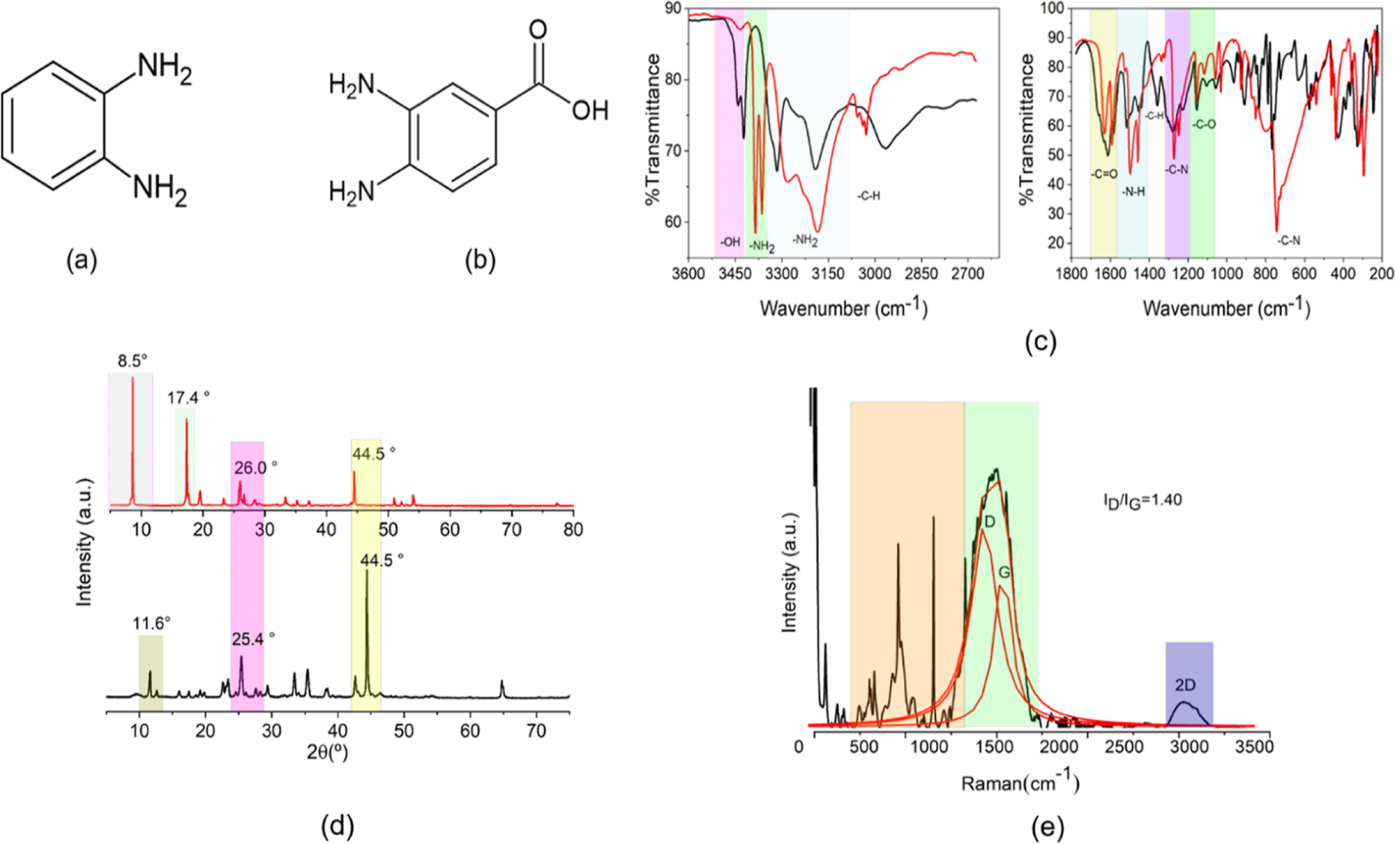
Chemical structure of the precursors used in the synthesis of *o*-OPDA (a) and 3,4-DABA (b). FTIR-ATR (c) spectra of CPDs-3,4-DABA (black line) and CPDs-OPDA (red line). Powder XRD (d) of CPDs-3,4-DABA (black line) and CPDs-OPDA (red line). Raman spectra (e) (black line) for CPDs-OPDA under 780 nm laser excitation. Gaussian deconvolution adjustment was made (red line).

To contribute to studies involving different CPD species and their cell viability, this work proposed the hydrothermal preparation of carbon nanoparticles from two similar organic molecules, *ortho*-phenylenediamine (*o*-OPDA) and 3,4-diaminobenzoic acid (3,4-DABA) (Figure 1a,b), via a domestic microwave. The characterization of carbon nanoparticles was available via structural, spectroscopic, and photophysical characterization. Cell viability and cytotoxicity mechanisms for both carbon nanoparticle species formed were also evaluated.

## 2 Results and Discussion

Recently, Silva and co-workers^36^ described the production of CDs from 3,4-DABA under different conditions using domestic microwave irradiation. Inspired by a similar diaminobenzene structure, the *o*-OPDA CDs were obtained under the same conditions.

First, a visual difference between the *o*-OPDA carbon nanoparticle and 3,4-DABA was observed during the synthesis (Supporting Information, Figures S1 and S2). Solid CPDs-3,4-DABA was obtained during the microwave irradiation, demonstrating a higher hydrophobicity characteristic because the solid sample was obtained during the synthesis, while the CPDs-OPDA was obtained as a yellow aqueous suspension after microwave irradiation. The solid CPDs-OPDA was obtained after drying under heating. Additionally, the CPDs-OPDA was purified using a chromatographic column.

In the literature, numerous studies have reported the synthesis of CDs from the *o*-OPDA precursor through different methodologies.^37−42^

Recently, Li et al. described the formation process of CDs from *o*-OPDA in the *o*-OPDA ethanolic solution by heating in a Teflon-lined stainless autoclave at 180 °C for 8 h. After purification by column chromatography, five compounds with yellow, green, and blue fluorescence emission were confirmed as CD mixtures and two molecular fluorophores named 2,3-diaminophenazine (DAP) and 2-amino-3-hydroxyphenazine (AHP).^43^ The same Li’s group prepared the CQDs from 3,4-DABA by a solvothermal procedure, heating the ethanolic solution of 3,4-DABA in a Teflon-lined stainless autoclave at 220 °C for 12 h.^44^

In our methodology, we analyzed the CPDs from *o*-OPDA and 3,4-DABA precursors based on their structural characterization. These dots show similar photoluminescence properties to CDs described in the literature.^43,44^ We used a domestic microwave with a short reaction time and a lower cost.

### 2.1 Structural Characterization

The attenuated total reflection Fourier transform infrared (FTIR-ATR) spectra, shown in Figure 1c, showed two peaks between 3384 and 3360 cm^–1^ that are attributed to −NH_2_/–OH stretching vibrations. Other −NH_2_ stretching vibrations were observed at 3283 and 3184 cm^–1^. In both regions, the −NH_2_ attribution can be correlated with open phenazine rings or terminal −NH_2_ groups present in the phenazine-2,3-diamine (2,3-DAP) polymer structure.^45^

The peaks at 1631 and 1587 cm^–1^ are assigned to the stretching vibration of −C=O bonds. Other peaks include 1492 and 1457 cm^–1^ for aromatic C=C bonds and −NH stretching, 1272 cm^–1^ for aromatic C–N bonds, 1242 cm^–1^ for aliphatic C–N bonds, and 747 cm^–1^ for the out-of-plane bending vibration of 1,2-disubstituted benzene rings. The FTIR results led us to believe that the CPDs-OPDA obtained under the experimental conditions could be the phenazine-2,3-diamine (2,3-DAP) polymer, similar to that obtained from the Ostrikov and Li groups.^46^

CPDs-3,4-DABA showed peaks between 3500 and 2800 cm^–1^ and 34420 and 3424 cm^–1^ attributed to stretching vibrations for the −NH_2_/–OH groups. The peaks at 3315 and 3192 cm^–1^ were characterized as −NH for the amide vibrational mode.^36^ In addition, the peaks at 1613 and 1581 cm^–1^, attributed to C=O, were attributed to amide I and II.^25,47^

In contrast to Li et al.,^44^ CQDs-3,4-DABA did not show carboxylic vibrations (C=O) in the 1705–1710 cm^–1^ range, indicative of carboxyl groups. This result reinforces the proposal of amide formation as a functional group in CPDs-3,4-DABA.

Unlike those of graphitic CDs, the powder XRD patterns of such CPDs-OPDA and CPDs-3,4-DABA systems consist of several intense sharp peaks, demonstrating their polycrystalline characteristics.

The polycrystallinity evidence showed the formation of polycyclic fragments during the carbonization of starting materials.^48^Figure 1d reports the powder XRD patterns from CPDs-OPDA and CPDs-3,4-DABA.

CPDs-3,4-DABA XRD shows a sharp diffraction peak present at 2θ ∼ 11° of graphene oxide^49^ and two peaks at 2θ ∼ 25 and ∼45° attributed to graphene.^50^ The XRD profile for CPDs-OPDA carbon nanoparticles showed similar peaks in addition at those at 2θ ∼ 8.5 and ∼17° in the function of the polymer crystalline structure.^51^ The XRD profiles of both carbon nanoparticles do not show graphitic peaks (∼25°), indicative of an amorphous structure, which can be seen in traditional carbonized CDs.^22^ In polymeric CDs, peaks appear from 20 to 40°, which means some other highly cross-linked polymer skeletons.^52^

The Raman spectrum for CPDs-OPDA carbon nanoparticles (Figure 1e) showed a prominent band between 1100 and 1700 cm^–1^ under 780 nm laser excitation. After the Gaussian deconvolution adjustment (Origin 18), peaks were observed for disordered graphite carbon sp^3^ at 1400 cm^–1^ (D band) and at 1538 cm^–1^ for graphitic structure sp^2^ (G band), in addition to the symmetric single peak 2D band at 2946 cm^–1^, confirming the graphene carbon structure.^53^ The Raman bands at 1274 cm^–1^ can be associated with shifting of the ν(C–N) for the phenazine structure.^54^ The bands at 762 and 1037 cm^–1^ are attributed to *o*-CN/–NH_2_ wag and ring deformation from the *o*-OPDA organic molecule.^55^

For CPDs-3,4-DABA,^36^ a typical G band at 1591 cm^–1^, a D band at 1388 cm^–1^, where the D band was only observed after the Gaussian deconvolution adjustment, and a 2D band at 2934 cm^–1^ were observed (Supporting Information, Figure S3a). Previously not discussed, peak D in CPDs-3,4-DABA presented a large area after deconvolution adjustment.^36^ Probably, this region has the contribution of a few sharp transitions at 1219–1231, 1365–1380, and 1458–1470 cm^–1^, attributed to N–H bending and C–N and C=N stretching, attributed to the aggregated structure of the organic compound.^44^

The scattering bands D and G explain the graphite void defects of sp^3^-hybrid amorphous carbon and the graphene layer of sp^2^-hybrid carbon atoms, respectively.^56^ The ratio of *I*_D_/*I*_G_ is related to the defect density of carbon and can give an idea of the extent of graphitization.^46,57^ Rogach et al. affirm that a small *I*_D_/*I*_G_ of ∼ 0.5 demonstrated a high crystalline CDs core, while larger ratios indicate a growing disorder and/or amount of amorphous carbon within CDs.^58^ The lower *I*_D_/*I*_G_ ratio for CPDs-3,4-DABA (*I*_D_/*I*_G_ = 0.86) than that for CPDs-OPDA (*I*_D_/*I*_G_ = 1.40) can be hypothesized to be due to the higher crystalline carbon core for CPDs-3,4-DABA than for CPDs-OPDA accompanied by an increase in N incorporation in CPDs-OPDA, which can create N-induced defects in the lattice.^37^

Thermogravimetric analysis differential thermogravimetry (TGA/DTG) was employed to intensify the understanding of the nanoparticle’s composition, focusing on the thermal events exhibited by CPDs-OPDA and CPDs-3,4-DABA. The TGA/DTG curves for CPDs-OPDA (Supporting Information, Figure S4a) revealed a primary thermal process beginning at approximately 110 °C, with a DTG peak at 141 °C. This event is predominantly associated with the loss of the −NH_2_ functional groups in the CPD structure, accounting for nearly 93% of its weight loss. For the precursor *o*-OPDA, a similar thermal event represents the sole pyrolysis step responsible for the compound’s thermal decomposition (Supporting Information, Figure S4b). After this main thermal event (around 145 °C), a second decomposition process commenced, marked by a DTG peak at 292 °C. This process is mainly due to the decomposition of the DAP polymer groups formed during synthesis, contributing to about 4% of the weight loss.^59,60^ The final pyrolysis process started at approximately 627 °C and consumed the remaining carbon cores entirely by the end of the TGA/DTG analysis at 900 °C.

The thermal behavior of CPDs-3,4-DABA, illustrated in the TGA/DTG curves (Supporting Information, Figure S4c), displayed four distinct thermal stages. The initial event, starting at an approximate temperature of 194 °C with a DTG peak at 205 °C, is attributed to the thermal decomposition of 3,4-DABA’s functional groups, leading to a weight loss of nearly 58%. This observation is corroborated by the similar thermal behavior observed in the TGA/DTG curves of the precursor 3,4-DABA (Supporting Information, Figure S4d), which undergoes a primary thermal decomposition process, resulting in 85% of its weight loss.^61^ For CPDs-3,4-DABA, a subsequent process began immediately after the conclusion of the main thermal event and extended up to 342 °C. This stage has a DTG peak at 336 °C and is related to the decomposition of the cross-linked polymer formed on the surface of CPDs-3,4-DABA, accounting for 6.2% of the weight loss.^62^ Then, the polymer backbone decomposes, reaching a DTG peak at 641 °C and contributing 11% to the weight loss.^63^ Lastly, the stage corresponding to the pyrolysis of the carbon core starts around 668 °C, leaving no residue by 900 °C.

Regarding solubility, the partition coefficients (log *P*_o/w_) were calculated for both carbon nanoparticles using the shake method with *n*-octanol and water. CPDs-3,4-DABA shows a more hydrophobic character (log *P*_o/w_ = 1.37) than CPDs-OPDA (log *P*_o/w_ = 0.54), confirming the visual observations during the synthesis. Probably, CPDs-3,4-DABA presents more lipophilic groups on the superficies state, in addition to more excellent cross-link conjugation.

**Figure 2:**
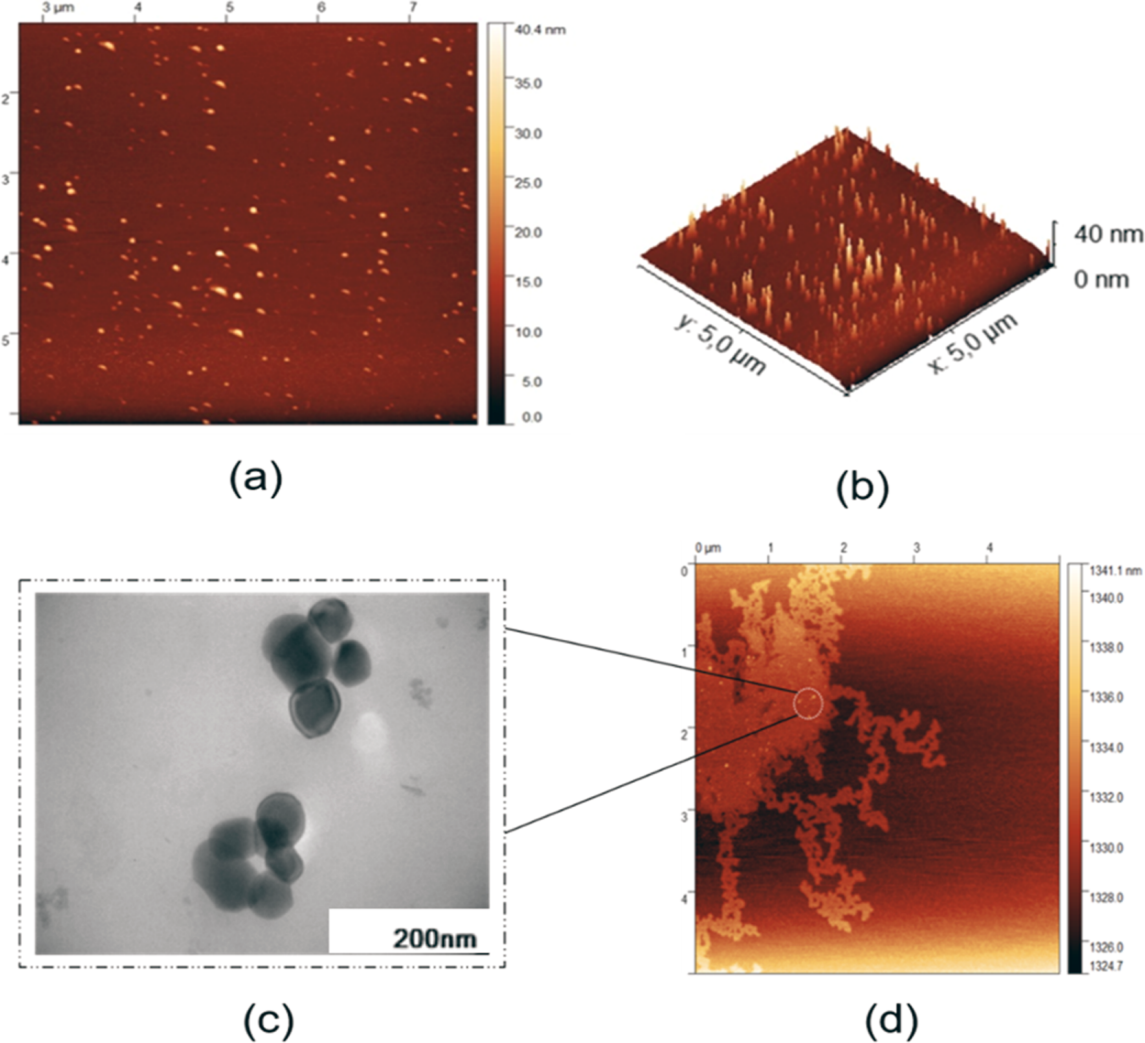
AFM images (a) and 3D distribution (b) of CPDs-3,4-DABA. TEM (c) and AFM (d) images of CPDs-OPDA.

Atomic force microscopy (AFM) images reveal that CPDs-3,4-DABA form spherical nanoparticles with an average particle size of 12.01 ± 2.10 nm (Figure 2a,b). As previously reported using transmission electron microscopy (TEM), CPDs-3,4-DABA showed agglomerated particles with large sizes between 28 and 39 nm.^36^

CPDs-OPDA in the TEM analysis (Figure 2c) presents the formation of agglomerates with a quasi-spherical shape, and the AFM image appearance is characteristic of a polymeric material with few dispersed nanoparticles (Figure 2d).

The AFM images were obtained after the centrifugation process to obtain more accurate dimensions of carbon nanoparticles (Supporting Information, Figure S5a–d). The nanoparticle sizes were 5.02 ± 1.03 nm for CPDs-OPDA and 14.01 ± 1.21 nm for CPDs 3,4-DABA (Supporting Information, Figure S3b).

The FTIR, XRD, and Raman results also indicated that CPDs-OPDA and CPDs-3,4-DABA synthesized in this work present a lower graphitization degree and an abundance of disordered polymer chains. Therefore, it could be further confirmed that the preparation of CPDs underwent the process of polymerization, cross-linking, and partial carbonization with carbon cores formed.

The authors^25−64^ suggest that the CPDs have a more complicated structure than CQDs. During the formation process of CPDs, the precursors, in general, small molecules and polymer precursors containing numerous amines, carboxyl, and hydroxyl groups,^37^ are dehydrated, polymerized, cross-linked, and carbonized, gradually forming the highly cross-linked and carbonized nuclei inside and polymer structures outside. The carbon core can exhibit several forms: an amorphous carbon core similar to CNDs, a latticed carbon core similar to CQDs, or dense carbon clusters formed by the carbonization of polymerized chains.

**Scheme 1:**
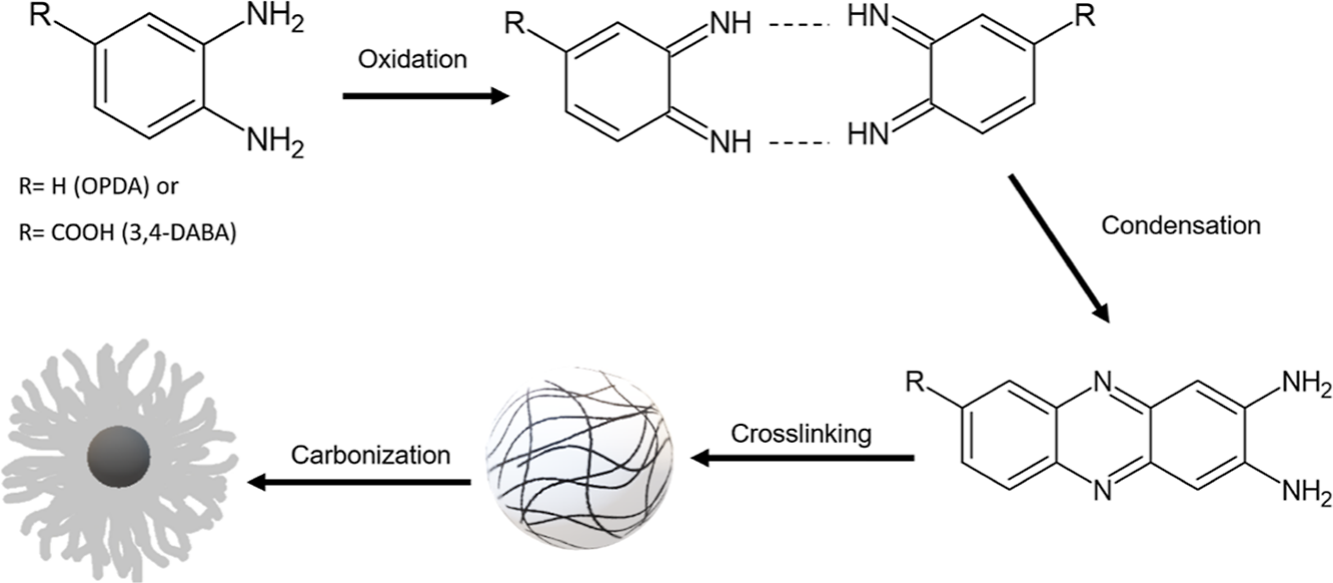
Proposed Mechanism of Formation of CPDs in a Domestic Microwave

Based on the proposal by Li et al.,^43^ Yang et al.,^52^ and Balachandran et al.,^37^ we can describe the mechanism (Scheme 1) involved in the formation of CPDs-OPDA; initially, a large amount of *o*-OPDA was oxidized and started dehydration in sequence condensation among functional groups, leading to the formation of extended molecular chains cyclized under hydrothermal conditions. With time, the phenazine-2,3-diamine (DAP) fluorophore polymer created small cross-linked polymer clusters. Subsequently, these clusters underwent a carbonization process. Then, C–N bonds formed in polymer fragments eventually led to rudiment carbon cores and cross-linked polymer chains being dominant rather than carbonized structures.

A speculative mechanism formation process from 3,4-DABA is shown in Scheme 1. However, the carboxyl groups can be polymerized with amine or hydroxyl groups between 3,4-DABA molecules to form the amide polymer, as observed in FTIR spectra. Furthermore, recently, Li et al.^44^ proposed that by introducing the carboxyl group in the phenylenediamine precursor, the —COOH group, can provide an acid environment and act as a catalyst for the formation of the molecular fluorophores.

Based on the FTIR and Raman results, we can infer that polymer chains retained the partial structure of precursors owing to incomplete carbonization, possessing carboxylic groups (O=C–O), pyridine N (=N), and amino groups (−NH_2_).

### 2.2 Spectroscopic Characterization

**Figure 3:**
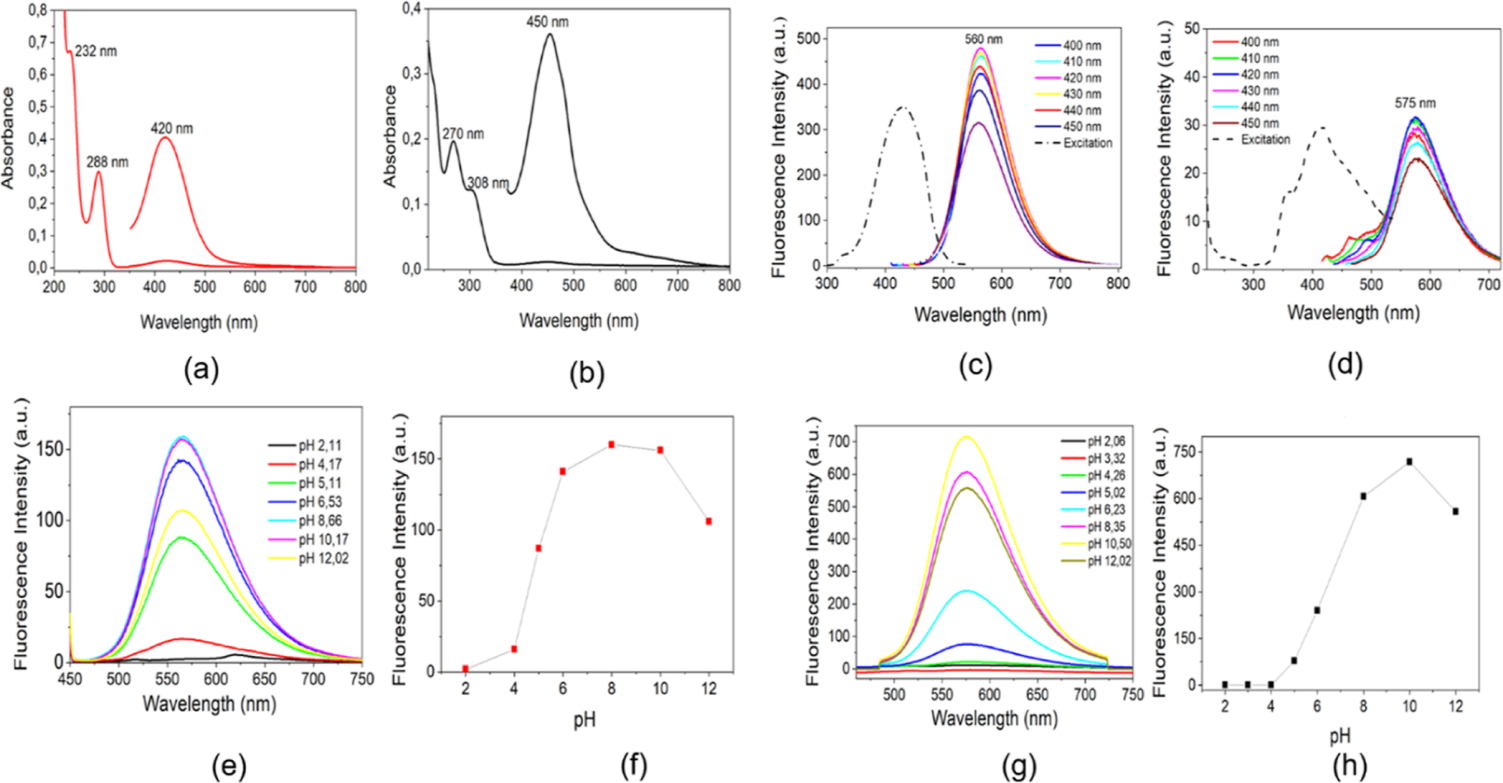
UV–visible absorption spectra of CPDs-OPDA (a) and CPDs-3,4-DABA (b) in aqueous solution. Fluorescence emission spectra under visible excitation of CPDs-OPDA (excitation/emission window 5/10) (c) and CPDs-3,4-DABA, (excitation/emission window 10/10) (d) in aqueous solution. Fluorescence emission versus pH plots for CPDs-OPDA (excitation/emission window 5/10) (e,f) and CPDs-3,4-DABA (g,h) (excitation/emission window 10/10).

The CPDs-OPDA spectra, in Figure 3a, showed absorption bands in the UV region at 232 nm, attributed to the *n*–σ* transition of C–NH or C–OH, while the absorption band at 288 nm is assigned to the π–π* and the 281 nm transition of the conjugated carbon core included C=C and C=N.^65^ The visible band at 420 nm is characterized by the molecular state transition from the conjugated structure to phenazine ring structure.^43^ As previously reported, CPDs-3,4-DABA presents absorption bands at 270 and 308 nm (Figure 3b), attributed to the π–π* of the conjugated carbon core and *n*–π* transitions of the surface state like C–O and C=O.^36^ For CPDs-3,4-DABA, the visible band at 450 nm for the molecular state transition shows a 30 nm bathochromic displacement from that for CPDs-OPDA, occasioned by introducing electron accepting groups (−COOH).^44^

The fluorescence emission from CPDs described in this work exhibits dual emission components in the UV and visible regions, which can be attributed to the core, surface, and molecular state.^37^ The blue fluorescence emission at the 290 and 340 nm regions for CPDs-OPDA is dependent on the excitation wavelength (Supporting Information, Figure S6a). In addition, for CPDs-3,4-DABA, when excited within the range 230 to 280 nm, the blue component (400 nm) was independent of excitation wavelength (Supporting Information, Figure S6b). Excitation-dependent emission is the most interesting property of CDs.^66^ The emission band in the UV region is identified as the core band, and originates from the sp^2^ carbon structure in the CPDs.^37^

The green component is reported by fluorescence emission with independent wavelength excitation for both carbon nanoparticles. The fluorescence spectra of CPDs-OPDA and CPDs-3,4-DABA (Figure 3c,d) show that the maximum emissions are at 560 and 575 nm, respectively. The Stokes shift was calculated using the maximum excitation wavelength, and the shift of 130 for CPDs-OPDA and 155 for CPDs-3,4-DABA was quite large compared to that of organic fluorophores. In this case, the origin of the green component is due to molecular fluorophore attachment to the carbon core.^37^

The photophysical characterization for CPDs-OPDA and CPDs-3,4-DABA was based on QY and fluorescence lifetime determination. The fluorescence QY for CPDs-OPDA and CPDs-3,4-DABA was calculated for the green components. Under visible excitation (λ_ex_ = 420 nm), QY = 19 and 2.98% for CPDs-OPDA and CPDs-3,4-DABA, respectively, using rhodamine 6G as the standard.

The resolved time fluorescence decay time from CPDs-OPDA and CPDs-3,4-DABA can be fitted to mono- and bi-exponential functions, respectively. The average lifetime (τ_AVE_) are described in Table 1.

**Table 1:** Fluorescence Lifetime for CPDs-OPDA and CPDs-3,4-DABA in Aqueous Solution at 295 K^a^

samples	TCSPC (λ_ex_= 440 nm, λ_em_= 550 nm)					
	τ (ns)	*A* (%)	⟨τ_AVE_⟩ (ns)	χ^2^	*k*_r_ (s^–1^)	*k*_nr_ (s^–1^)
CPDs-OPDA	1.83	100	1.83	1.287	1.04 × 10^8^	4.43 × 10^8^
CPDs-3,4-DABA	1.082.23	64.0135.99	0.75^a^	1.202	3.85 × 10^7^	1.29 × 10^9^

a τ_AVE_: average lifetime of the biexponential function was computed as described in eq S1 (Supporting Information eq S1).^67^

The fast lifetime components (τ_1_) observed in the both CPDs are dependent on the nonradiative relaxation process from the trap state of the core to the surface state. The second component (τ_2_) observed only for CPDs-3,4-DABA indicated the emission from the intrinsic core band, produced by the graphitic carbon core.^37^

The transition rate constants, radiative constant (*k*_R_), and nonradiative constant (*k*_R_) were^33,68^ calculated according to the equation
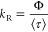
where Φ is the QY of the CDs and ⟨τ⟩ is the average fluorescence lifetime of the CDs.

The *k*_NR_, which is the sum of the internal conversion and intersystem crossing rate constants, was calculated by the following equation
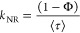


The order of magnitude *k*_NR_ for CPDs-OPDA was 10 times lower than that for CPDs-3,4-DABA. The increase in lifetime is related to a decrease in the nonradiative relaxation process, justified by changes in the surface state between nanoparticles.^33^ The lower *k*_NR_ value suggested that CPDs-OPDA possesses an efficient recombination process occasioned by strong coupling of the excited core state with the surface state.^68^ The higher lifetime and %QY obtained for CPDs-OPDA have been described for CDs where the N-doping occurs.^68^ Furthermore, the oxygen-containing functional groups in the CPDs-3,4-DABA structure contribute to the formation of various energy gaps.^37,69^

Considering the application of carbon nanoparticles in biological medium, it was necessary to evaluate the fluorescence profile versus pH plot.

The fluorescence intensity of emission at ∼560 nm from CPDs-OPDA increases intensively in the pH interval 2–12 (Figure 3e,f). Similar results were obtained from the CPDs-3,4-DABA fluorescence emission ∼ 575 nm response versus pH plot (Figure 3g,h).

In general, the fluorescence emission from CDs vs pH can be justified based on the protonation–deprotonation of the amine/imine and carboxyl group on the surface of CDs.^70^ Furthermore, the tendency to form aggregates is a factor that contributes to the decrease in fluorescence intensity with pH.^71^

Both carbon nanoparticles, when dissolved in ultrapure water, resulted in pH = 4.70 and 6.70 for the CPDs-3,4-DABA and CPDs-OPDA aqueous suspensions, respectively. Under these conditions, their surface charges given by the zeta potential were −3.90 mV at (CPDs-3,4-DABA) and −0.9 mV (CPDs-OPDA). The observed pH is associated with the p*K*_a_ values of functional groups present on the carbon nanoparticle surface and in the surface-bound fluorophores. The p*K*_a_ values of the carboxylic groups (p*K*_a_ ≈ 4–5) and the pyridine nitrogen (p*K*_a_ ≈ 4–6) are lower than those of primary amine (NH_2_) groups (p*K*_a_ ≈ 9–11) or phenolic OH groups (p*K*_a_ ≈ 8–10) as well as amide C(=O)(NH) groups (p*K*_a_ ≈ >8.5).^72^ The pH and zeta potential results revealed that CPDs-OPDA presents more basic groups on the surface than CPDs-3,4-DABA.

### 2.3 Biocompatibility and Cell Death Mechanism

**Figure 4:**
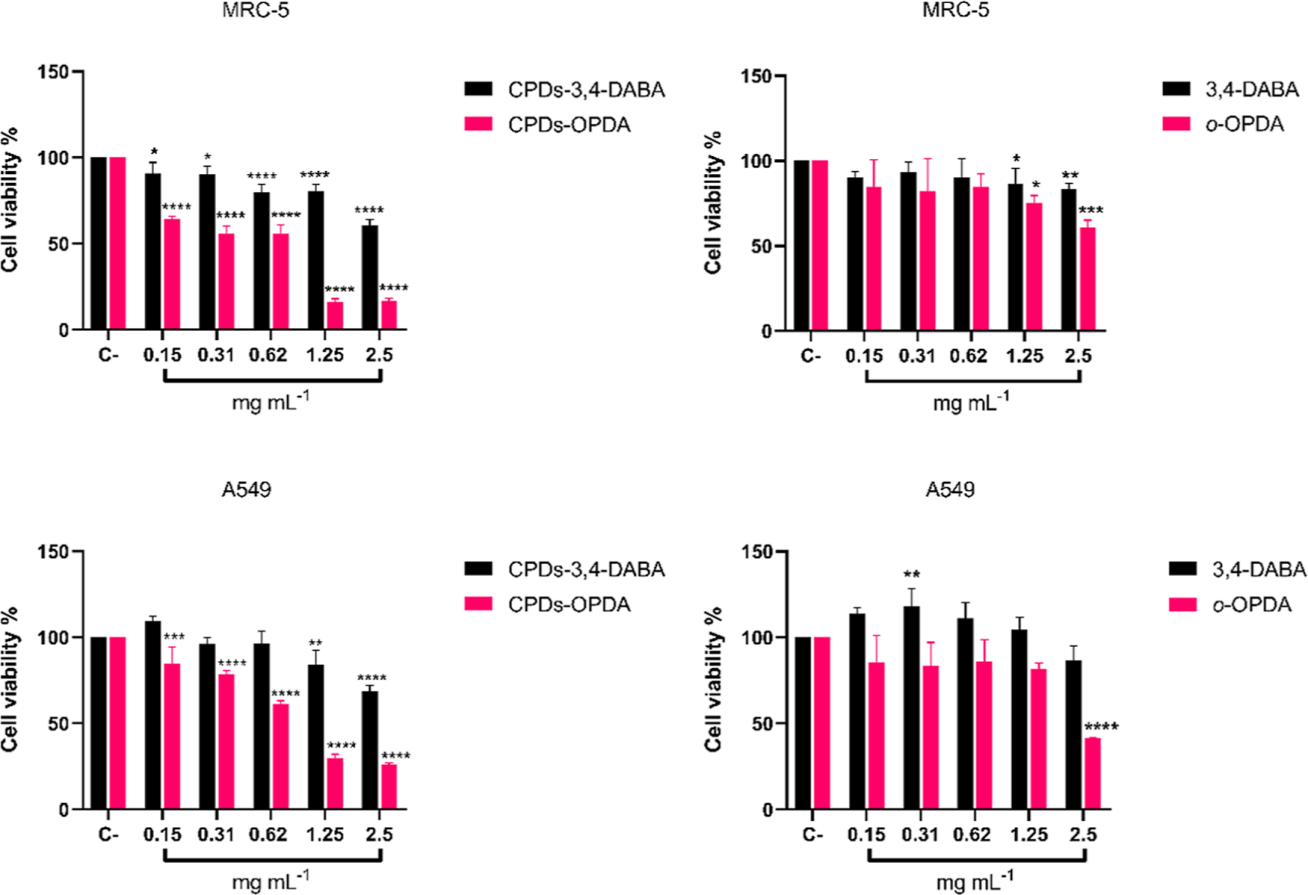
CPDs-3,4-DABA and CPDs-OPDA and their carbogenic precursor’s (*o*-OPDA and 3,4-DABA) effects, as assessed by the MTT assay in MRC-5 and A549 cells after 24 h treatment. Values represent the mean ± SE. The percentage results were compared with the negative control (C−). (*p < 0.05, **p < 0.01, ***p < 0.001, and ****p < 0.0001.).

The biocompatibility of the CPDs-OPDA and CPDs-3,4-DABA carbon nanoparticles on lung cells was analyzed by using a 3-(4,5-dimethylthiazol-2-yl)-2,5-diphenyltetrazolium bromide (MTT) assay. The results showed that both carbon nanoparticles significantly reduced cell viability in both lung cell lines, A549 and MRC-5, compared to the negative control group (C−), especially at the highest concentration (1.25–2.5 mg mL^–1^) after 24 h incubation. In contrast, the carbonaceous precursors 3,4-DABA and *o*-OPDA also decreased cell viability, although to a lesser extent than for the CPDs-3,4-DABA and CPDs-OPDA carbon nanoparticles. Notably, in the MRC-5 cell line, the viabilities at the highest concentration of 3,4-DABA and *o*-OPDA precursors were 83 and 60%, respectively, whereas CPDs-3,4-DABA and CPDs-OPDA showed viabilities of 60 and 16%, respectively. Moreover, for the A549 tumor cell line, the viabilities at the highest concentration of 3,4-DABA and *o*-OPDA precursors were 86 and 41%, respectively, and for CPDs-3,4-DABA and CPDs-OPDA, the viabilities were 68 and 26%, respectively. This suggests that the observed cytotoxicity is mainly attributed to CPDs-3,4-DABA and CPDs-OPDA carbon nanoparticles rather than their precursors (Figure 4).

The CPDs-OPDA fluorescence was observable through confocal microscopy and fluorescence imaging. From this observation, we can conclude that CPDs-OPDA is internalized by the cells and spreads throughout all cells, indicating successful uptake (Supporting Information, Figure S7). On the other hand, CPDs-3,4-DABA does not produce sufficient fluorescence to be visualized in confocal microscopy images (data not shown).

The lower confocal visualization from CPDs-3,4-DABA can be discussed based on its higher hydrophobic character than that of CPDs-OPDA, as previously reported for the partition logarithm (log *P*_o/w_). Furthermore, CPDs-3,4-DABA showed a lower QY than CPDs-OPDA under visible fluorescence excitation.

**Figure 5:**
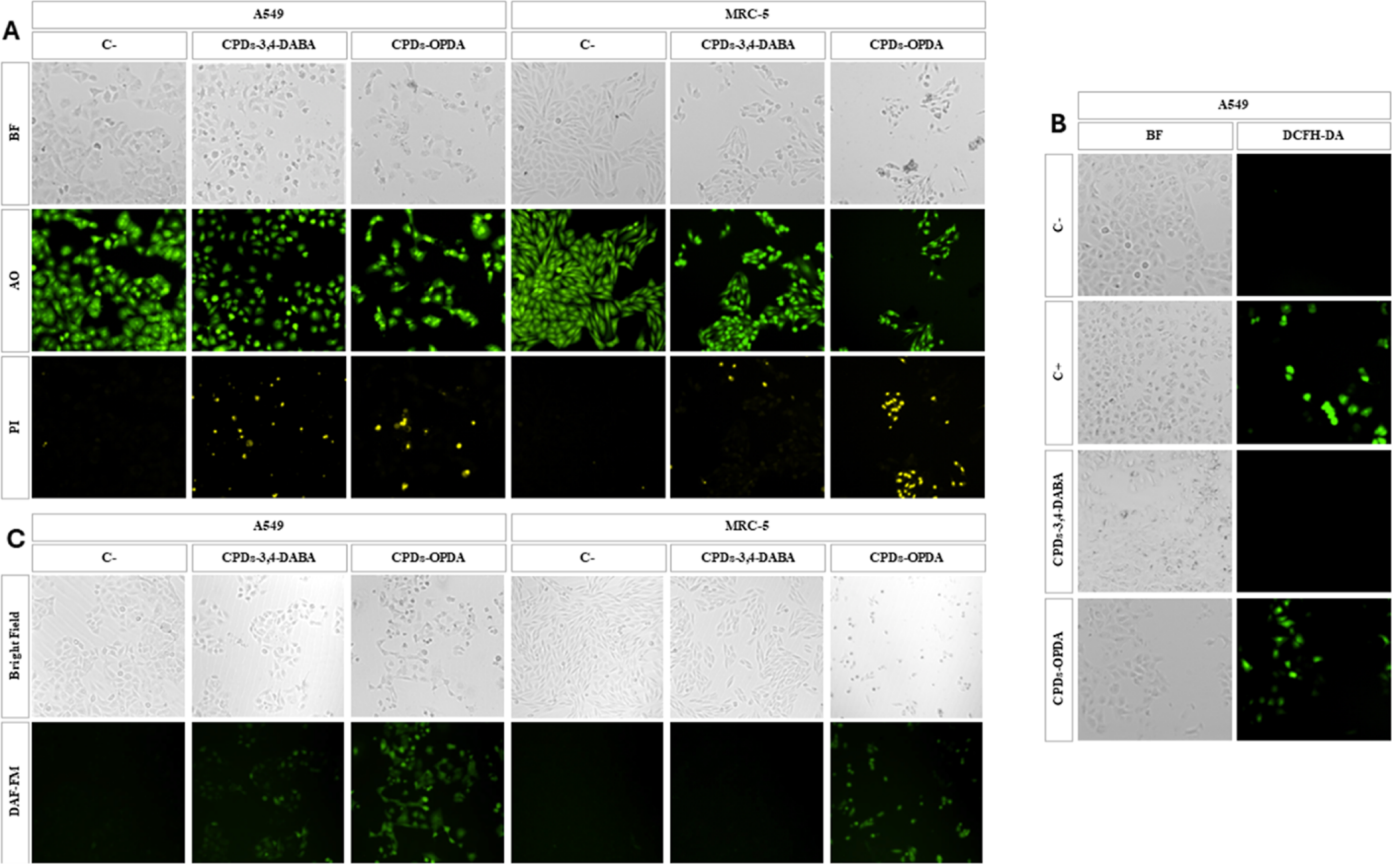
Microscopic images of MRC-5 and A549 cells after 24 h of treatment with CPDs-3,4-DABA and CPDs-OPDA (1.25 mg mL^–1^), stained with a dual dye (AO/PI). (C−) Untreated control cells, observed under a magnification of 100×. Abbreviations: AO (acridine orange) and PI (propidium iodide) (A). Fluorescence microscopy of ROS production in MRC-5 and A549 cells after 1.5 h of treatment with CPDs-3,4-DABA and CPDs-OPDA (1.25 mg mL^–1^); as the positive control (C+), hydrogen peroxide (1000 μmol L^–1^) was used (B). Fluorescence microscopy of NO production in MRC-5 and A549 cells after 24 h of treatment with CPDs-3,4-DABA and CPDs-OPDA (1.25 mg mL^–1^) (C).

Given the evaluation of cellular morphology, a fluorescence staining assay using the dyes acridine orange (AO) and propidium iodide (PI) was conducted, which not only facilitated the detailed observation of cellular morphology but also enabled the assessment of cell viability through membrane integrity (Figure 5a). AO is a dye that stains both live and dead cells, whereas PI only stains cells with compromised membrane integrity.^73^ After 24 h of treatment with CPDs-3,4-DABA or CPDs-OPDA, a reduction in cell density was evident, particularly in the cells treated with CPDs-OPDA. Furthermore, an increase in PI-labeled cells, indicating cellular death, was observed in both CPDs-3,4-DABA- and CPDs-OPDA-treated cells.

Cellular cytotoxicity can be initiated through oxidative stress, often induced by nanoparticles.^74^ This process involves the formation of reactive oxygen species (ROS), leading to oxidative damage and, ultimately, cell death.^75^ In our study, A549 lung tumor cells were exposed to CPDs-3,4-DABA and CPDs-OPDA for 1.5 h. Following this treatment, the cells underwent staining with diacetyl dichlorofluorescein (DCFH-DA) and were observed under a fluorescence microscope.

The results demonstrated that only CPDs-OPDA showed green fluorescence emission characteristics of ROS production, while CPDs-3,4-DABA did not lead to intracellular ROS generation (Figure 5b). These results suggested that CPDs-OPDA increased the levels of ROS in the A549 cells.

Nitric oxide (NO) can be generated in the presence of ROS in response to inflammatory conditions.^76^ To investigate intracellular NO generation following treatment with CPDs-3,4-DABA and CPDs-OPDA, we performed staining of A549 and MRC-5 cells using 4-amino-5-methylamino-2′,7′-difluorescein (DAF-FM) and analyzed them through fluorescence microscopy after 24 h of treatment. The results presented in Figure 5c demonstrate an increase in NO intracellular production in the A549 cells treated with both CDs-3,4-DABA and CPDs-OPDA. In contrast, in MRC-5 cells, only the treatment with CPDs-OPDA led to a noticeable elevation in NO levels, while CPDs-3,4-DABA treatment alone did not show a significant effect. However, for CPDs-OPDA, green fluorescence emission was more evident in both MRC-5 and A549 culture cells than for CPDs-3,4-DABA. Juang et al. demonstrated the cytotoxic effects of six different CNDs. Specifically, CND-PA and CND-PB enhanced inflammation through increased NO production, a mechanism attributed to the higher number of amino groups present in their structures.^75^ However, CDs with more carboxyl and hydroxyl groups induced cell proliferation.^77^

To justify why CPDs-3,4-DABA did not lead to the intracellular generation of ROS, the determination of the DPPH^•^ free radical’s action as an antioxidant was proposed. Barbosa et al. showed that CDs from lemon exhibited higher antioxidant capacity than CDs from *o*-OPDA.^78^ These results can be associated with more oxygenated surface groups in the lemon CDs.^79^ These groups act as hydrogen donors to react with unstable free radical molecules.^80^ Moreover, CPDs-3,4-DABA has a more conjugated sp^2^ carbon core than CPDs-OPDA, as observed in Raman spectra. The radical scavenging mechanism in CDs involves electron transfer to the sp^2^ CDs core.^81^

CPDs-3,4-DABA demonstrated higher antioxidant activity, approximately 38%, than CPD-OPDA, 11% (Supporting Information, Figure S8).

Therefore, the lower cytotoxicity of CPDs-3,4-DABA can be attributed to the lack of ROS generation and limited cellular uptake, as observed by confocal microscopy. This absence of ROS production leads to less oxidative stress, while the limited internalization means fewer interactions with intracellular structures, thereby reducing the potential for cytotoxic effects. Furthermore, the higher antioxidant capacity of CPDs-3,4-DABA likely contributes to its ability to neutralize ROS, further mitigating oxidative stress and cytotoxicity. These combined factors explain the observed differences in cytotoxicity between CPDs-3,4-DABA and CPDs-OPDA.

## 3 Conclusions

Factors such as solvent, carbonization time, and temperature in the production of CDs have well established their influence on the structural and photoluminescence characterization of these carbon nanoparticles. However, in this work, it was shown that alteration of a substituent group in the organic molecule can influence the cross-linking degree and carbonization mechanism of CDs. It is worth mentioning that the characterization of the UV–visible profiles and fluorescence emission is not always sufficient to determine the classification of CDs. The carbon nanoparticles obtained in this work by the domestic microwave irradiation method from the carbogenic precursor *o*-OPDA and 3,4-DABA were classified as polymer dots (CPDs). The structural characterization results involving FTIR and Raman spectra, XRD patterns, and AFM images indicated that both have a polymer cross-linked under carbon core associated with functional groups from the precursor. Furthermore, CDs-3,4-DABA presents a more organized core region than CPDs-OPDA. The different carbon nanoparticle species showed similar UV–visible profiles and fluorescence emission. However, there are different photophysical mechanisms. CPDs-OPDA exhibited higher QY values and a longer fluorescence lifetime. The decrease in cell viability in normal and tumor cells in the presence of CPDs-OPDA may be associated with the production of ROS. The lower CPDs-3,4-DABA cytotoxicity has been associated with an increase in antioxidant activity compared to that in CPDs-OPDA. CPDs obtained in this work can act as ROS generating or/and scavenging properties depending on the starting material and have shown much potential in biomedical applications.

## 4 Materials and Methods

*o*-OPDA, 3,4-DABA, rhodamine 6G (R6G), 2,2-diphenyl-1-picrylhydrazyl (DPPH), and silica gel high-purity grade, pore size 60 Å, 200–400 mesh particle size were from Sigma-Aldrich. For cell studies, the following reagents were utilized: MTT, Dulbecco’s modified Eagle’s medium (DMEM), AO, PI, DCFH-DA, phenol red, and DAF-FM, all of which were purchased from Sigma-Aldrich. All of the other reagents were used as received without further treatment. Moreover, ultrapure water (deionized water, 18.2 MΩ cm) was used throughout the experiments.

### 4.1 Synthesis of Carbon Nanoparticles

Carbon nanoparticles were fabricated via domestic microwave (Midea, 700 W, 2450 MHz)-assisted irradiation using 3,4-DABA and *o*-OPDA as the carbogenic source.

CPDs-3,4-DABA and CPDs-OPDA carbon nanoparticles were obtained similarly to that described by Silva et al.^36^ The CPDs-OPDA preparation was based on dissolving 0.15 g of *o*-OPDA in 50:50 (v/v) water and absolute ethanol (99.5%). Subsequently, the resulting solution was subjected to domestic microwave irradiation for 8 min. The CPDs-OPDA brown solution was purified by column chromatography via elution with acetonitrile (AC).^43,82^ All fractions were collected and analyzed by UV–visible and emission fluorescence profiles (Supporting Information, Figure S9). The five fractions showed similar absorption and emission profiles. In this case, the five fractions were pooled and treated as a single sample. The fractions were evaporated in the drying oven to obtain the solid and then submitted for the second stage: purification utilizing a 0.20 μm pore syringe filter (Kasvi). Subsequently, the solution was left in the drying oven to obtain the purified yellow–brown solid.

### 4.2 Characterization

FTIR spectra were acquired on a Cary 630 spectrometer (Agilent Technologies) using the ATR accessory for the wavenumber range 4.000–950 cm^–1^. Raman spectra were acquired with a LabRAM HR Evolution spectrometer (Horiba). The zeta potential analysis was performed on a Zetasizer Nano Instrument (Malvern Analytical). UV–vis–NIR absorption spectra were measured on a Cary 5000 spectrometer (Agilent Technologies). Fluorescence emission spectra and excitation spectra were recorded on a Cary Eclipse (Agilent Technologies). The fluorescence decay curves were recorded on a combined fluorescence lifetime and steady-state spectrometer (F900) using a time-corrected single photon counting system (Horiba). Powder XRD was performed by a German Bruker D8 Focus XRD with a graphite monochromatized Cu Kα radiation source (*k* = 1.54056 Å). TGA was performed by the thermal analyzer DTG-60H (Shimadzu) and TGA 55 (TA Instrument). The morphology and size of the CD dispersion were characterized by AFM, which was carried out using scanning by a probe using a specific Scanning model SPM-9600 (Shimadzu) and characterized by a transmission electron microscope operated at 100 k (Hitachi HT-7700). The aqueous suspension sample was deposited on a carbon-coated copper grid. Confocal imaging was performed using a TCS SP8 confocal microscope (Leica Microsystems). The zeta potentials of the samples were measured by light scattering techniques on a Litesizer DLS 700 (Anton Paar). The samples were measured at room temperature. The zeta potentials were then calculated by applying the Smoluchowski relation, which is valid for aqueous solutions containing low electrolyte concentrations.

### 4.3 Fluorescence QY Measurements

Fluorescence QY determination was performed by comparing the results obtained using an indirect method.^83^ The QY from CPDs-3,4-DABA and CPDs-OPDA in an aqueous solution was determined using λ_exc_ = 420 nm. Rhodamine 6G (dissolved in ethanol; QY = 0.95)^84^ was used as the QY standard solution for visible wavelength excitation. The QY calculation was done using the following eq 1
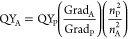
1where the subscript “P” refers to standard solutions and “A” refers to sample solutions of CPDs-3,4-DABA and CPDs-OPDA. Grad is the area of integrated fluorescence emission spectra, and *n* is the refractive index of the solvent (*n* = 1.33 and 1.36 for water and ethanol, respectively).

### 4.4 Carbon Nanoparticle Fluorescence Emission in pH Buffer Solution

A series of pH Britton–Robison (BR) buffer solutions were prepared from an equal mixture of 0.1 mol L^–1^ acetic acid, 0.1 mol L^–1^ boric acid, and 0.1 mol L^–1^ phosphoric acid.^85^ The pH values in the range of 2–12 were adjusted with the addition of 1 mol L^–1^ NaOH.

### 4.5 Determination of the Partition Coefficient (log *P*_o/W_)

The partition coefficient value was obtained from the shaken flask method.^86^ A 0.002 g amount of CPDs-OPDA (or CPDs-3,4-DABA) was added into a mixed solution containing 5 mL of water and 5 mL of *n*-octanol. The system was then stirred for 24 h, and after this period, the different phases were collected. The fluorescence emission spectra were obtained under λ_exc_ = 420 nm for each phase. The log *P*_o/w_ was calculated using the ratio of the integral area of each emission spectra in *n*-octanol and water (Supporting Information, Figure S10) following eq 2
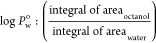
2

### 4.6 Thermogravimetric Analysis

Thermogravimetric analyses of CPDs-OPDA or CPDs-3,4-DABA were carried out in a Thermal Analyzer TGA 55 (TA Instruments). For analysis, about 5 mg of carbon nanoparticles in the solid state was heated in an aluminum sample holder between 30 and 900 °C, at a heating rate of 5 °C min^–1^, under a nitrogen atmosphere at a flow rate of 60 mL min^–1^.

### 4.7 AFM Analysis

The samples for AFM analysis were prepared under different conditions. Initially, solid CPDs-OPDA or CPDs-3,4-DABA was suspended in water and dispersed on a mica support. For the second sample, the solid (concentration 0.1 mg mL^–1^) was suspended in water and centrifuged under 1000 rpm for 5 min, where 5 μL of the supernatant solution was dispersed on the mica.

### 4.8 Cell Line and Culture

The MRC-5 nontumoral lung cells and the A549 lung cancer cells were cultured in DMEM containing 10% fetal bovine serum (FBS), l-glutamine (2 mmol L^–1^), and the antibiotics penicillin (100 μg mL^–1^) and streptomycin (100 μg mL^–1^). These cells were maintained in a humidified incubator at 37 °C and 5% CO_2_.

#### 4.8.1 Cell Viability Assay—MTT Method

The cytotoxicity effects of CDs on the lung cells were evaluated using the MTT reagent according to Mosmann,^87^ with modifications. Cells were plated (1 × 10^4^ cells/well) in sterile 96-well plates and incubated in a humidified incubator (37 °C and 5% CO_2_). After 24 h, different concentrations of CPDs-OPDA and CPDs-3,4-DABA, as well as the carbonaceous precursors 3,4-DABA and *o*-OPDA, (ranging from 2.5 to 0.15 mg mL^–1^) were added to the wells for an additional 24 h under the same culture conditions as those described earlier. All samples tested were solubilized in dimethyl sulfoxide (DMSO). The concentrations used in the assays were prepared in culture medium, with a maximum concentration of 1% DMSO.

After the incubation period, the supernatant was removed, and a solution of 0.5 mg mL^–1^ MTT was added for 4 h. Then, the solution was removed, and the formazan crystals formed during the period were solubilized in DMSO. Absorbance was measured using an ELISA plate reader at a wavelength of 540 nm. Statistical analysis was performed using GraphPad Prism version 8.0 (analysis of variance followed by Dunnett’s post-test).

#### 4.8.2 Uptake Assay by Confocal Imaging

Cells were plated (1 × 10^4^ cells/well) in sterile 10-well black plates and incubated in a humidified incubator (37 °C and 5% CO_2_). After 24 h, CPDs-OPDA (1.25 mg mL^–1^) was added to the wells for an additional 24 h under the same culture conditions as those described earlier. After the incubation period, the medium was removed, the medium without phenol red was added to the wells, and then, images were captured using a Leica TCS SP8 confocal microscope. During image acquisition, the wavelengths were 488 nm for excitation and 590 nm for emission. The confocal microscopy settings were consistent for all samples, and images were acquired at 400× optical zoom.

#### 4.8.3 AO and PI Dual Staining Assay

Cells were seeded onto 24-well plates (0.5 × 10^5^/well) and incubated at 37 °C with 5% CO_2_ for 24 h. They were then treated with CPDs-OPDA or CPDs-3,4-DABA for 1.5 h (1.25 mg mL^–1^). Afterward, a dual fluorescent staining solution containing 10 μg mL^–1^ AO and 10 μgmL^–1^ PI was added and allowed to incubate at 37 °C for 40 min. The cells were washed twice with PBS and cultured in a phenol red-free medium for subsequent fluorescence microscopy analysis using a Nikon Eclipse Ti microscope at 100× optical zoom.

#### 4.8.4 Intracellular ROS Detection

Cells were seeded onto 24-well plates (0.5 × 10^5^/well) and incubated at 37 °C with 5% CO_2_ for 24 h. They were then treated with CPDs-OPDA or CPDs-3,4-DABA for 1.5 h (5 mg mL^–1^). Afterward, 50 μmol L^–1^ DCFH-DA was added and allowed to incubate at 37 °C for 40 min. The cells were washed twice with PBS and cultured in a phenol red-free medium for subsequent fluorescence microscopy analysis using a Nikon Eclipse Ti microscope at 100× optical zoom. As a positive control, cells were treated with hydrogen peroxide at a concentration of 1000 μmol L^–1^ for 1.5 h.

#### 4.8.5 NO Measurement with DAF-FM Diacetate

Cells were seeded at a density of 1 × 10^5^ cells per well in sterile 12-well plates and cultured at 37 °C with 5% CO_2_ for 24 h. Following this, the cells were treated for 24 h with 1.25 mg mL^–1^ CPDs-OPDA or CPDs-3,4-DABA. After the incubation period, the cells were washed with PBS and incubated in a culture medium without FBS and phenol red with 5 μmol L^–1^ DAF-FM for 30 min at 37 °C. Subsequently, the cells underwent another round of washing with PBS, and the culture medium was replaced with a phenol red-free medium for fluorescence microscopy analysis using a Nikon Eclipse Ti microscope. The images were captured at 100× optical zoom.

#### 4.8.6 Evaluation of the Antioxidant Capacity

The antioxidant capacities of CPDs-OPDA and CPDs-3,4-DABA were evaluated by a colorimetric assay based on the discoloration of the oxidized form of DPPH violet to the reduced form (yellow color). 250 μL of a 100 μmol L^–1^ DPPH solution dissolved in methanol was mixed with carbon nanoparticles in DMSO solution at 50 and 1000 μg mL^–1^. The absorbance at 517 nm was measured after a 30 min agitation in the dark.^88,89^ The DPPH inhibition percentage was calculated using formula 3
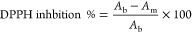
3where *A*_b_ is the absorbance of the blank and *A*_m_ is the absorbance of the corresponding solution at the indicated wavelength.^88^

## Supporting Information Available

Images of solutions during carbonization via domestic microwave irradiation of both CPDs; Raman spectra and TEM images of CPDs-3,4-DABA; TGA/DTG curves of both precursors and CPDs; AFM images of both CPDs and the corresponding distribution height profile; fluorescence emission spectra under UV excitation of both CPDs; confocal microscopy imaging of cellular uptake of the fluorescent CPDs-OPDA; UV–visible spectra for both CPDs in the presence of scavenging DPPH^•^ radicals; chromatographic column image of CPDs-OPDA; UV–vis absorption spectral fractions of CPDs-OPDA; and fluorescence emission spectra for log *P* and equation τ_AVE_ (PDF)
