# Supplementary material for: Carbonized Polymer Dots: Influence of the Carbon Nanoparticle Structure on Cell Biocompatibility
Source: ACS Omega. 2024 Sep 5;9(37):38864–77. doi: 10.1021/acsomega.4c05011 (PMC11411664; doi:10.1021/acsomega.4c05011)
Supplement: Supplementary file 1 — ao4c05011_si_001.pdf [file ao4c05011_si_001.pdf]

Supplementary Material

Carbonized Polymer Dots: influence of carbon nanoparticle structure in the cell biocompatibility

Mayara Martins Caetano[1,2], Amanda Blanque Becceneri[3], Marcos Vinícius Ferreira[1,2], Rosana Maria Nascimento Assunção[1,2], Roberto Santana da Silva[3] and Renata Galvão de Lima*[1,2]

[1] *Instituto de Química, Universidade Federal de Uberlândia, Avenida João Naves de Ávila, 2121 - Bairro Santa Mônica, Uberlândia, MG, Brazil*
[2] *Instituto de Ciências Exatas e Naturais do Pontal, ICENP, Universidade Federal de Uberlândia, Rua Vinte, 1600, 38304-402, Tupã, Ituiutaba, MG, Brazil.*
[3] *Faculdade de Ciências Farmacêuticas de Ribeirão Preto, USP, Avenida do Café s/n, Vila Monte Alegre, Ribeirão Preto, SP 14040-903, Brazil*

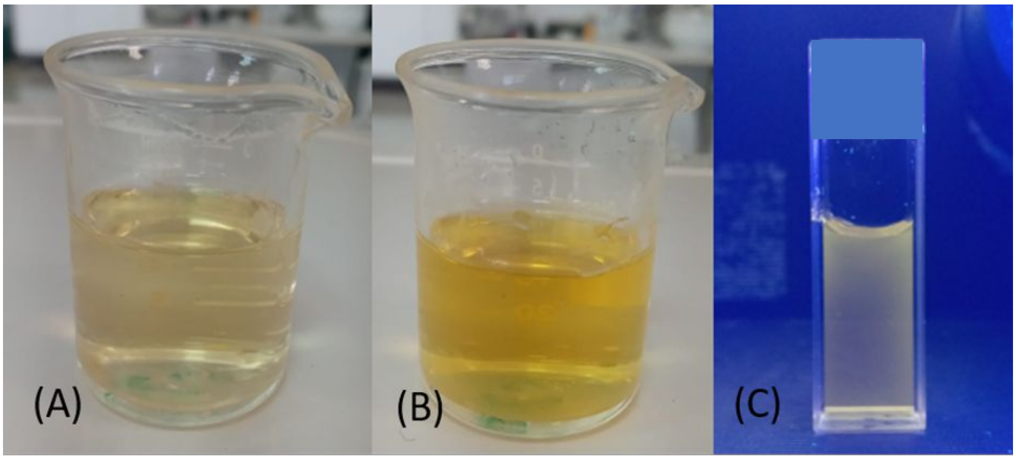

Figure S1. *o*-OPDA solutions before carbonization (A), after carbonization via domestic microwave irradiation (B), and under UV light excitation (365 nm) (C).

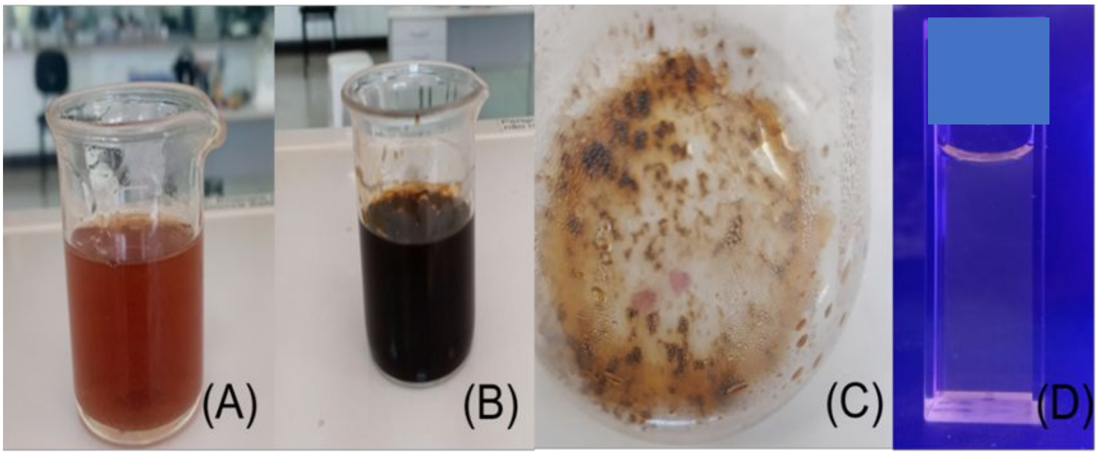

Figure S2. 3,4-DABA solutions before carbonization (A), during carbonization via domestic microwave irradiation (B), the formation of the solid after carbonization (C) and under the influence of UV light (365 nm) (D).

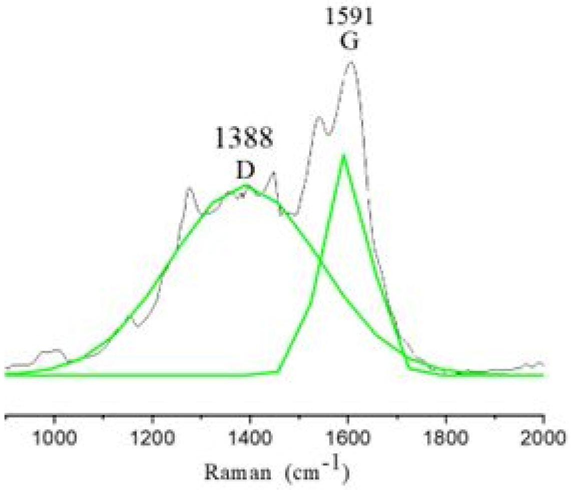

(a)

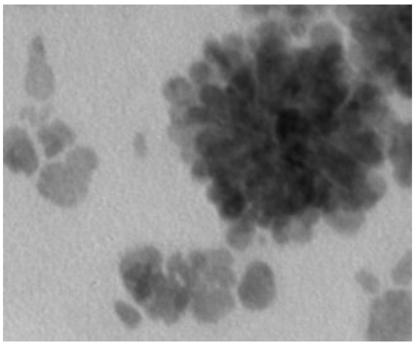

100 nm

(b)

Figure S3. Raman spectra (**a**) (black line) for CPDs-3,4-DABA under 780 nm laser excitation. Gaussian deconvolution adjustment (green line). TEM images of CPDs-3,4-DABA (**b**).

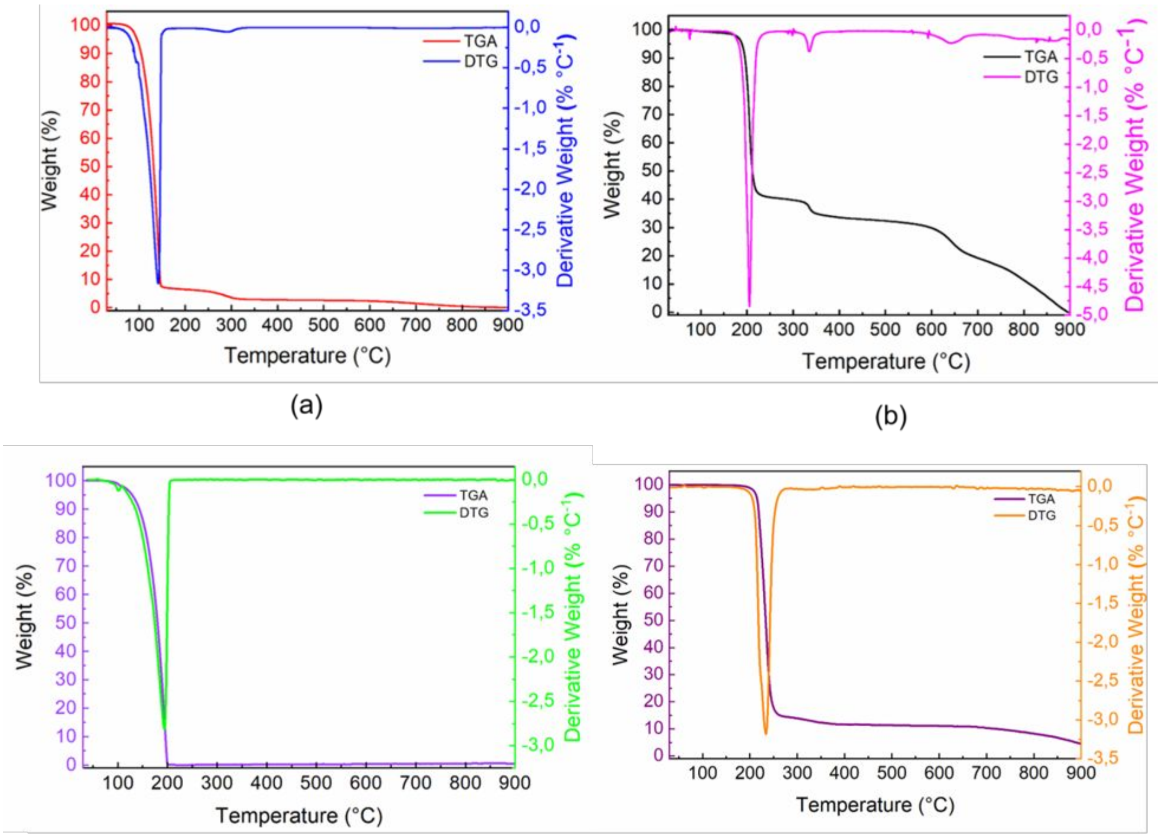

(a) (b)

(c) (d)

Figure S4. TGA/DTG curves of *o*-OPDA carbon nanoparticle (a), CPDs-3,4-DABA (b), precursor *o*-OPDA (c) and 3,4-DABA (d).

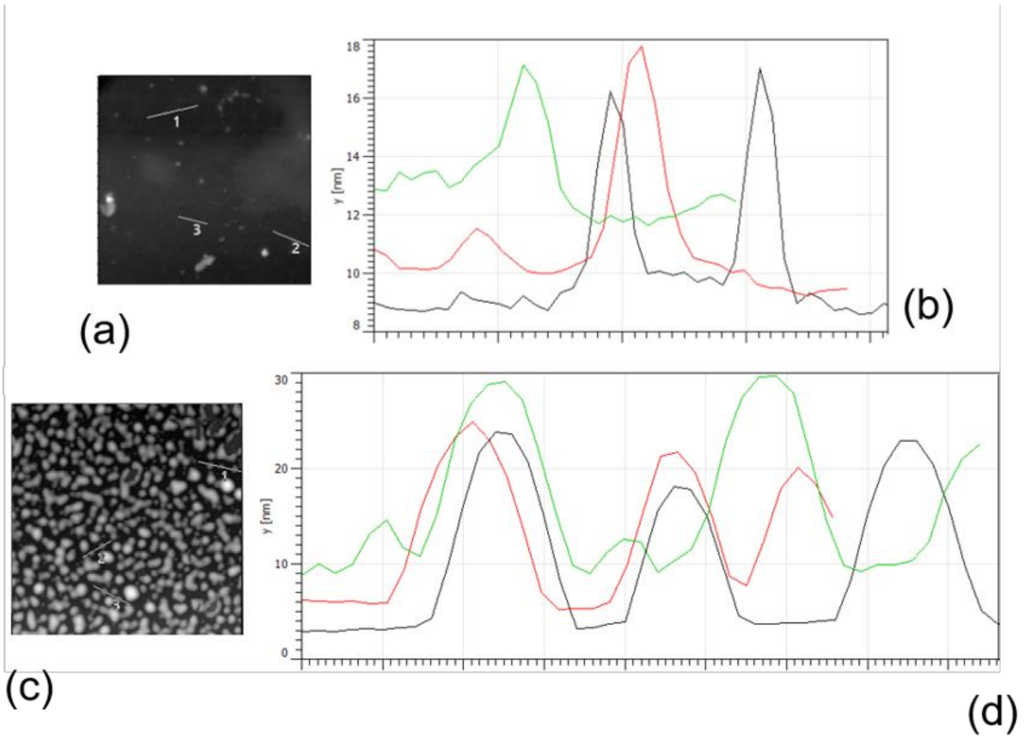

Figure S5. AFM images of the CPDs-OPDA (a) and CPDs-3,4-DABA (c). The corresponding distribution height profile along the white line in (b) and (d) for the as-prepared CPDs-OPDA (b) and CD 3,4-DABA (d), respectively.

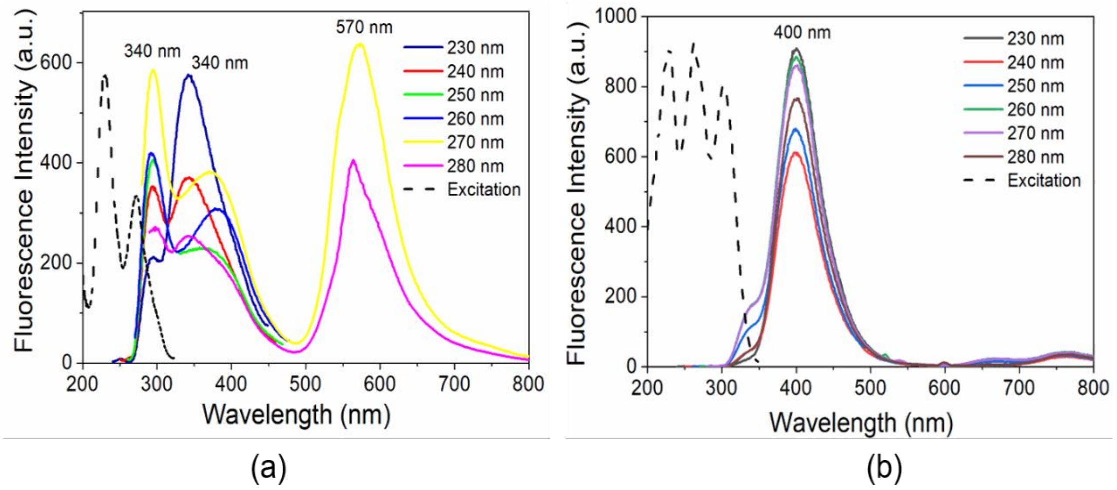

Figure S6. Fluorescence emission spectra under UV excitation of CPDs-OPDA (excitation/emission window 10/10) (a) and CDs-3,4-DABA (excitation/emission window 5/5) (b) in aqueous solution.

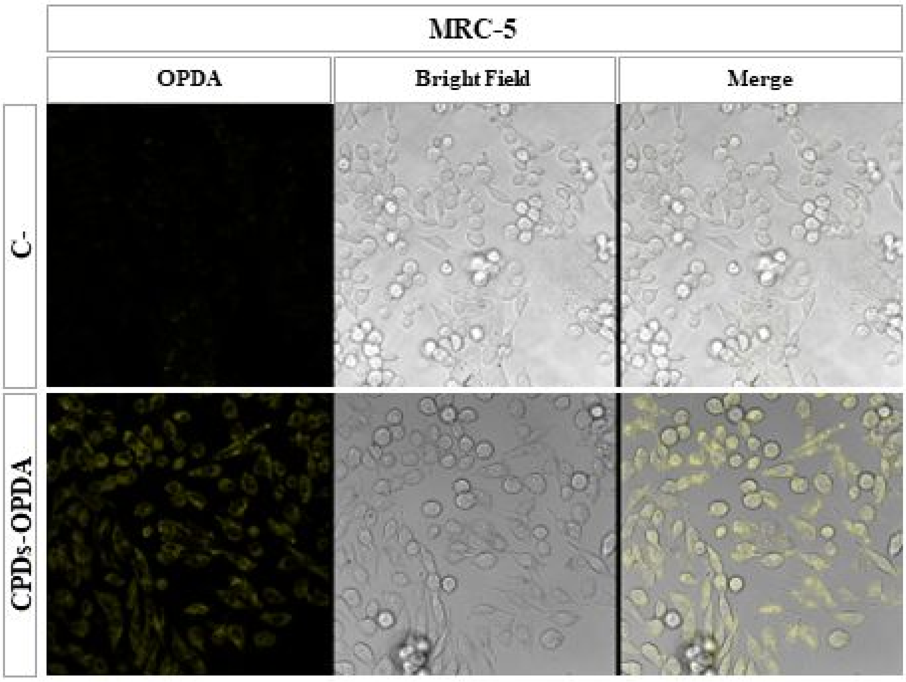

Figure S7. Confocal microscopy imaging of cellular uptake of the fluorescent CPDs-OPDA in MRC-5 cells incubated for 24 h at 0.07 mg mL^{-1}. The shown images are representative of one of the triplicates. 400× total magnification, with excitation at 488 nm and emission at 590 nm.

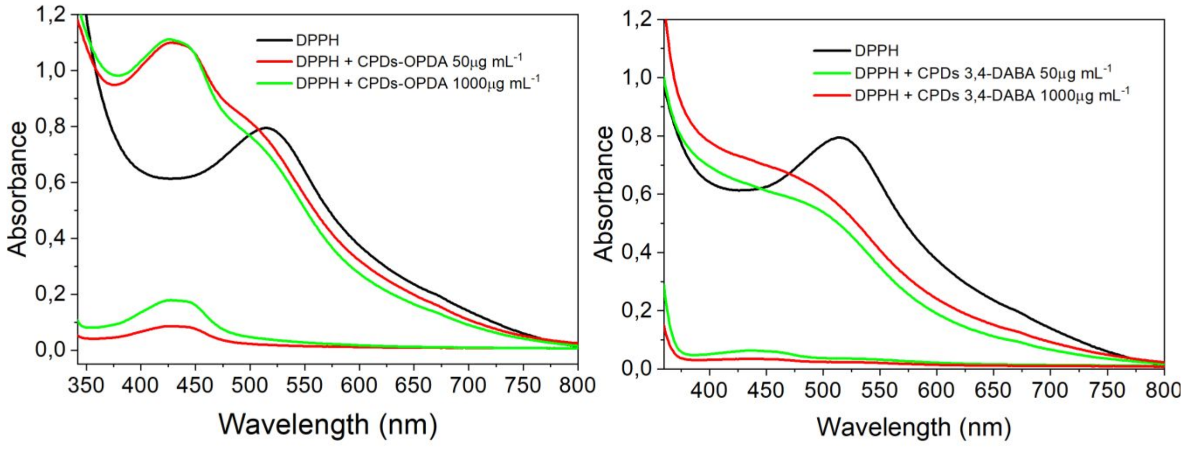

(a) (b)

Figure S8. UV–visible spectra for CPDs-OPDA (a) and CPDs-3,4-DABA (b) dissolved in DMSO and in the presence of scavenging of DPPH• radicals.

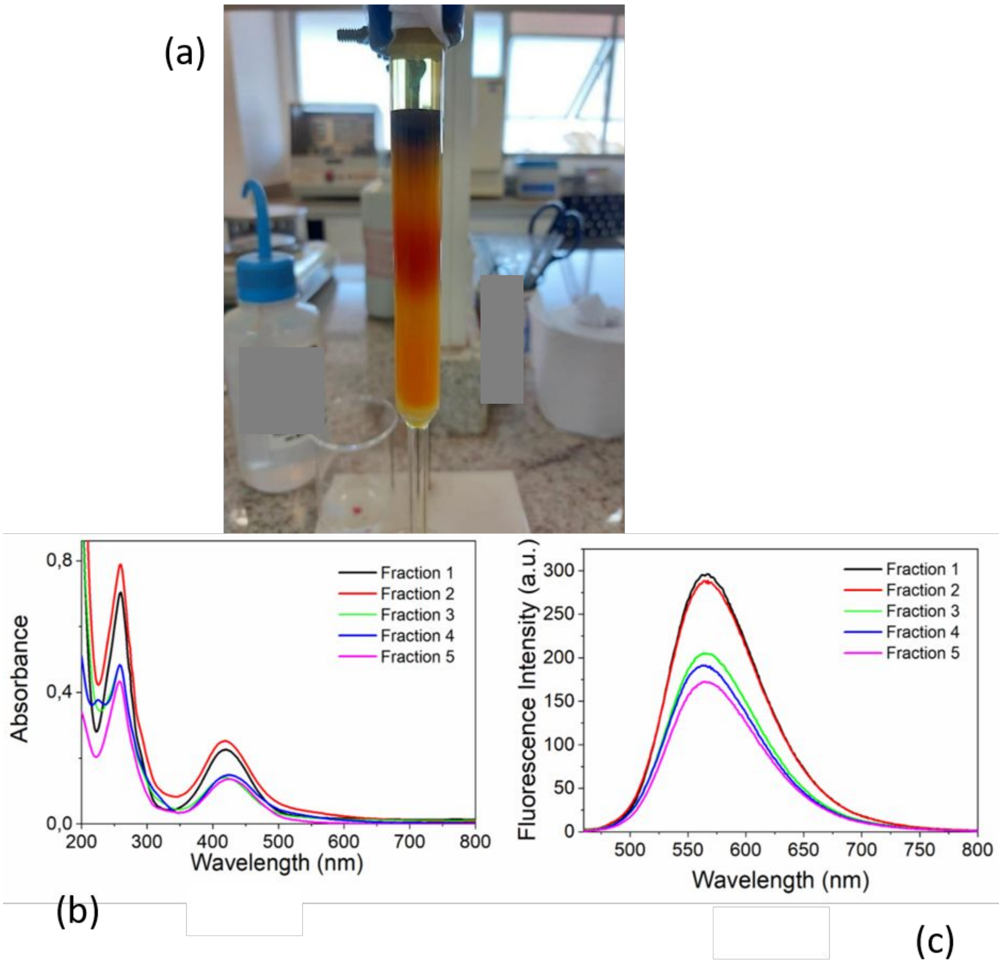

Figure S9. Chromatographic column image of CPDs-OPDA (a). UV-Vis. absorption spectra (b) and fluorescence emission spectra ($\lambda_{excitation}$=420 nm) of the column chromatography fractions of the CPDs-OPDA (c). Fraction 1: 100% acetonitrile. Fractions 2, 3, 4 and 5: 80% acetronitrile:20% water.

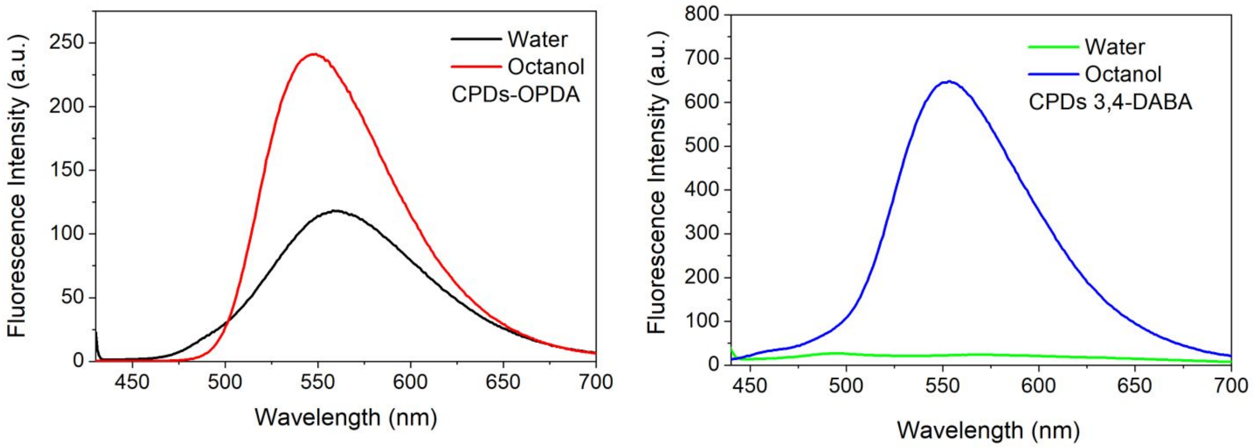

Figure S10. Fluorescence emission spectra in visible excitation of CPDs-OPDA (a) and CPDs 3,4-DABA (b) dissolved in water and ethanol (under UV wavelength excitation 5/10).

Equation S1:

$$\tau_{AVE} = \frac{(A_1\ x\ \tau_1)2 + (A_2 x \tau_2)2}{(A_1 x \tau_1 + A_2 x \tau_2)}$$
